# Determining Subclinical Cardiovascular and Cardiac Diseases in Patients with Non-Alcoholic Fatty Liver Disease

**DOI:** 10.5152/tjg.2022.22075

**Published:** 2023-03-01

**Authors:** Beliz Bahar Karaoğlan, Cansın Tulunay, Çağlar Uzun, Elif Peker, Nil Özyüncü, Zeynep Ellik, Diğdem Kuru, Sibel Turhan, Berna Savaş, Ayşe Erden, Ramazan Idilman

**Affiliations:** 1 Department of Gastroenterology, Ankara University Faculty of Medicine, Ankara, Turkey; 2 Department of Cardiology, Ankara University Faculty of Medicine, Ankara, Turkey; 3 Department of Radiology, Ankara University Faculty of Medicine, Ankara, Turkey; 4 Department of Pathology, Ankara University Faculty of Medicine, Ankara, Turkey; 5 Department of Biostatistics, Ankara University Faculty of Medicine, Ankara, Turkey

**Keywords:** Atherosclerosis, cardiovascular disease, myocardial dysfunction, non-alcoholic fatty liver, non-alcoholic steatohepatitis

## Abstract

**Background:**

The aims of the present study were to determine the subclinical coronary atherosclerosis and myocardial dysfunction in patients with non-alcoholic fatty liver disease (NAFLD), who were asymptomatic for cardiac disease.

**Methods:**

A total of 61 non-alcoholic fatty liver disease patients were enrolled in the study. The 10-year probability of cardiovascular events was evaluated according to the pooled cohort equation risk score (atherosclerotic cardiovascular disease). The coronary artery calcium score was measured. Conventional echocardiographic examination was followed by 2- and 3-dimensional speckle tracking echocardiography.

**Results:**

Patients with non-alcoholic steatohepatitis had significantly higher insulin resistance (*P = .*018), serum alanine aminotransferase (*P = .*002) and aspartate aminotransferase levels (*P = .*021), hepatic steatosis (*P = .*023), and fibrosis (*P = .*001) than non-alcoholic fatty liver disease patients.

The mean Atherosclerotic Cardiovascular Disease score was 7.5% ± 6.9% and 37% of the patients had medium and high cardiovascular disease risk. Cardiovascular disease (>1) was found in 30% of the patients. Interestingly, 56% had significant and extended atherosclerotic plaques. Among the patients with moderate-to-high atherosclerotic cardiovascular disease scores, 63% had significant atherosclerotic plaques and 21% had extensive plaque burden. The presence of non-alcoholic steatohepatitis did not significantly affect cardiovascular risk.

Non-alcoholic steatohepatitis was deleterious on left ventricle diastolic functions. Mean A velocity in non-alcoholic steatohepatitis patients was significantly increased compared to non-alcoholic fatty liver disease patients (87.0 ± 17.5 cm/s vs. 72.3 ± 13.6 cm/s, *P = .*002). Mean E/e’ ratio was 8.1 ± 2.0. Submyocardial fibrosis detected had a slightly higher occurrence in non-alcoholic steatohepatitis patients than in non-alcoholic fatty liver disease patients (*P = .*530).

**Conclusion:**

NAFLD seems to be associated with an increased risk of subclinical cardiovascular disease and myocardial dysfunction in asymptomatic patients with cardiac disease.

## Introduction

Non-alcoholic fatty liver disease (NAFLD) is now the most common chronic liver disease in Western countries and in Turkey.^1,2^ Non-alcoholic fatty liver disease encompasses a wide spectrum of hepatic conditions ranging from simple steatosis as NAFL to steatohepatitis (NASH), cirrhosis, and hepatocellular carcinoma.^1,2^ Non-alcoholic fatty liver disease is a multisystem disease that affects a variety of extrahepatic organ systems.^2,3^ There is cross-talk between liver and heart.^3-8^ Non-alcoholic fatty liver disease is closely associated with obesity, diabetes mellitus, and metabolic syndrome, all of which are established risk factors for cardiovascular disease(CVD).^2-4^ Previous studies demonstrated an association between NAFLD and increased CVD events.^5-8^ This association is less evident in CVD-related or overall mortality.^8^

Studies have shown that NAFLD is associated with an increased risk of subclinical atherosclerosis including increased carotid intima-media thickness (CIMT), coronary artery calcium (CAC), arterial stiffness, and endothelial dysfunction.^5-8^ Cardiovascular risk (CVR) is predicted by standard risk prediction methods such as the Systematic Coronary Risk Evaluation or the Pooled Cohort Equation Atherosclerotic Cardiovascular Disease (ASCVD) risk calculators.^4,8^ The latter is widely used to determine the 10-year risk of coronary artery disease (CAD). Coronary artery calcium is a sensitive and specific predictor to screen for early atherosclerosis.^9,10^ Increased calcification levels are indicative of a high total plaque burden and increased risk for prevalent CAD.^9,10^

Non-alcoholic fatty liver disease may cause altered left ventricle (LV) structure and function and develop diastolic heart failure and cardiac dysfunction.^11-13^ Both 2D and 3D speckle tracking echocardiography can be used to measure LV strain during the early diagnosis of structural and functional changes in the heart.^11-13^ Previous studies demonstrated that NAFLD was increased by 3 times the risk of mild-to-moderate LV diastolic dysfunction independently of other CVR factors.^12,13^ Cardiac magnetic resonance imaging (MRI) is a non-invasive imaging technique with high sensitivity and specificity. It provides detailed information about the morphology, myocardial structure, and tissue perfusion and function.^14,15^ Images of T1 mapping are obtained using the modified look-locker imaging (MOLLI) technique.^14,15^

In contrast to previous studies that evaluated the association between NAFLD and CVD in individuals with NAFLD diagnosed by abdominal sonography or computed tomography (CT), the present study aimed to determine the subclinical coronary atherosclerosis and myocardial dysfunction in biopsy-proven NAFLD patients, who were asymptomatic for cardiac disease.

## Materials and Methods

This is a prospective cross-sectional single-center study including biopsy-proven NAFLD patients, in the Ankara University School of Medicine, Department of Gastroenterology, Liver Diseases Outpatient Clinic between July 2017 and June 2019. The diagnosis of NAFLD was based on biochemical, radiological, and histological criteria, excluding other forms of acute and chronic liver diseases. Data were collected from outpatient visit charts. All patients provided their personal and familial medical history at the time of their recruitment into the study. Written informed consent was obtained from each patient. The study protocol conformed to the ethical guidelines of the 1975 Declaration of Helsinki and was approved by the local ethical committee of the Ankara University School of Medicine.

Biochemical and serological tests were conducted in the central laboratory of Ankara University School of Medicine. Laboratory tests were performed at the time of diagnosis, during the follow-up period, and during exacerbation of liver enzyme levels.

### Hepatic Steatosis and Liver Stiffness Measurement

All patients underwent abdominal sonography confirming the presence of fatty infiltration of the liver. Hepatic steatosis and liver stiffness were measured using a Vibration Controlled Elastography (VCTE) FibroScan 502 touch (Echosens, Paris, France) with an M or XL probes with different body build types. The VCTE measured the controlled attenuation parameter (CAP) (dB/m) and liver stiffness (kPa) simultaneously.

All liver biopsy specimens were evaluated by 1 pathologist blinded to the clinical and biochemical data. Histological features of the samples were interpreted using the criteria of Kleiner et al.^16^

### Cardiac Evaluation

The ASCVD risk score was calculated for all participants according to the 2013 American College of Cardiology/American Heath Association Guideline on the Assessment of CVR by using the online calculator http://www.cvriskcalculator.com/.^17^ Those with a 10-year risk of <5% were considered low risk, those between 5% and 7.5% were borderline risk, 7.5%-20% were medium risk, and those of >20% were considered high risk.^18^ The ASCVD risk score does not apply to patients under the age of 40.^17,18^

Coronary CT was conducted by a 64-slice CT scanner (Toshiba, Aquillon 64, Toshiba Medical Systems, Otowara, Japan). The CT data were transferred to a remote workstation (Vitrea 2, Vital Images, Plymouth, Minn, USA) for post-processing and subsequent evaluation. The CAC score was determined using the dedicated software Vitrea 2. The CAC scores were calculated applying the Agatston method, by summing calcium scores of each artery where coronary calcification was identified as a lesion, with an area greater than 1 mm^2^ and a peak intensity greater than 130. Computed tomography images were acquired with the following parameters: tube current of 300 mA, tube voltage of 120 kV, tube rotation time of 350 ms, section thickness of 3 mm, field of view of 200-220. All scans were analyzed by an experienced radiologist (CU). The CAC score was categorized into 0,1 to 10,11 to 100,101 to 400, and >400. Coronary artery calcification is defined as CAC > 1.^9,10,19^

The echocardiography was conducted by experienced cardiologists (ST, CT, and NO) using GE Vivid E95, with a 4 MHz 4V sector probe (General Electric, Horten, Norway). Standard measurements of chambers and diastolic parameters were performed following related recommendations.^20^ Both 2D and 3D images were recorded for speckle-tracking echocardiography. By using a fully sampled 4 MHz matrix array transducer, the full‐volume acquisition was recorded from the cardiac apex for 3 consecutive cardiac cycles during a single breath-hold. The echocardiographic images were analyzed by a cardiologist (CT) blinded to the clinical condition of the subjects. The 2D global longitudinal strain (GLS) was measured using the 2D speckle-tracking Automated Function Imaging (AFI) (Echopac, GE Vingmed, Horten, Norway). The offline analysis of 3D speckle-tracking was performed using the Echopac 4D Auto LVQ software package (GE Vingmed). First, the operator designated the long axis of LV in 3 different apical views (4, 3, and 2 chambers). This software distinguishes the endocardial border of LV and tracks the border for an entire cardiac cycle. Finally, by using the standard 16‐segment model, the curves of peak strain are determined. The recorded 3D Speckle-Tracking Echocardiography (STE) parameters were LV end‐diastolic volume, end‐systolic volume, stroke volume, ejection fraction (EF), GLS, global circumferential strain (GCS), global radial strain (GRS), area strain, twist in degrees, and torsion degrees per second.

The most important indicators of LV diastolic dysfunction are deterioration in LV relaxation (velocity(é) decrease), decreased LV refilling forces, increased LV diastolic stiffness, especially an increase in LV end-diastolic pressure (increase in E/é ratio).^21^ E(early)/late(A) diastole peak velocity ratios are an indicator of LV diastolic dysfunction. The reference value of the E/A ratio is in the range of 0.8-2.E/A, ratio < 0.8 indicates increased filling pressure.^21^ The ratio of early trans-mitral velocity over the average of tissue Doppler-derived septal and lateral early-diastolic velocity (E/e’) is a determinant of LV filling pressures. The normal value of the E/e’ ratio is <8; the range of 8-14 was considered undetermined, and >14 is increased LV filling pressure that was considered to indicate abnormal diastolic function. Automated Function Imaging-derived 2D GLS measurement, an indicator of LV systolic functions, as well as 3D GLS, GCS, and GRS measurements, was calculated. The normal average GLS value is approximately −20%, but reference values might vary depending on the vendor. Global longitudinal strain values above −18% are generally considered normal, and the probability of systolic dysfunction increases when absolute values fall below −16% (e.g., −14% indicates a worse strain value than −18%).^20^ Myocardial performance index (MPI) evaluates LV systolic and diastolic functions together, with a normal value of 0.39 ± 0.05, which is expected to be increased in the case of impaired LV functions.^22,23^ However, there is no normal range indicated in the guidelines for strain analysis of 3D speckle-tracking measurements.

Cardiac MRI was performed using Aera 1.5 Tesla Siemens (Erlangen, Germany). T1 mapping was conducted with the original MOLLI sequence (MOLLI 3(3)3(3)5). The variant (MOLLI 5(3)3) was acquired and followed by T2 mapping. Sequence parameters for MOLLI 5(3)3 were TE/TR/flip-angle:1.12 ms/279.8 ms/35°; voxel size 1.4 × 1.4 × 10 mm, bSSFP; GRAPPA: 2; half scan (7/8 Fourier). For the MOLLI 3(3)3(3)5, the parameters were TE/TR/flip-angle:1.64 ms/418.64 ms/50°; voxel size: 1.7 × 1.7 × 10 mm, bSSFP; GRAPPA: 2; half scan (6/8 Fourier). For standard T1 sequence, 1009 ± 59(±2 SD)ms and Siemens T1 sequence 1006 ± 56 (±2 SD)ms were accepted as mean reference values.

### Definitions

The primary aim was to determine the subclinical coronary atherosclerosis and myocardial dysfunction in patients with NAFLD by comparing them with the NAFL and NASH patients.

Obesity was defined based on the World Health Organization criteria using the body mass index (BMI) of 25-29.9 kg/m^2^ defining overweight and ≥30 kg/m^2^ defining obesity.^24^ Insulin resistance (IR) was calculated based on fasting plasma glucose and insulin values using the homeostasis model assessment (HOMA) and IR method.^25^ Percentage of body fat score was determined using a Tanita body composition analyzer.

### Statistical Analysis

Means, standard deviations, medians, ranges, and frequencies, and percentages were used for descriptive statistics. Comparisons between 2 groups were performed with Student’s *t*-test or Mann–Whitney *U* test depending on the distribution of the data. Categorical variables were assessed with the chi-square test or Fisher’s exact test. Correlations between continuous and/or ordinal variables were calculated using Pearson’s correlation or Spearman’s rho analysis, where applicable. A *P*-value of less than .05 was considered statistically significant.

### Results

#### Patients

A total of 61 NAFLD consecutive patients were recruited for the study. The median age was 54 years (range: 33-67 years), and they were predominantly females (66%).The median BMI was 30.4 kg/m^2^ (range: 19.6-43.1 kg/m^2^), with 34.4% of the patients being overweight (25-29.9 kg/m^2^) and 55.7% obese (≥30 kg/m^2^). Body fat ratio was 33.5% ± 8% (range: 15.3%-49.6%). Among the patients, 42.6% had diabetes mellitus, 36.1% had hypertension, 21.3% had hyperlipidemia, 9.8% had been treated with statins, 9% were ex-smokers, and 5% were active smokers. The mean systolic and diastolic blood pressure was 134.4 ± 16.5 mmHg and 79.9 ± 8.1 mmHg, respectively. The mean waist circumference was 103.9 ± 11.0 cm and the hip circumference was 111.5 ± 10.6 cm. At the time of diagnosis, the median serum alanine aminotransferase (ALT), aspartate aminotransferase (AST), and gamma-glutamyl transpeptidase (GGT) levels were 41 U/L (range: 6-215 U/L), 31 U/L (range: 11-132 U/L), and 47 U/L (range: 12-204 U/L), respectively. The median total bilirubin level was 0.7 mg/dL (range: 0.3-2.7 mg/dL), and the median international normalized ratio was 0.95 (range: 0.8-1.2). The baseline characteristics of the patients are shown in Table 1.

At baseline histopathological evaluation, the median NAFLD activity score was 5 (range: 2-8) and fibrosis was 1 (range: 0-3) (Table 2). The VCTE valuation indicated the mean CAP and liver stiffness values at 316.5 ± 47.8 dB/m (median: 316 dB/m, range: 218-400 dB/m) and 7.6 ± 5.3 kPa (median:6 kPa, range:3.5-34.7 kPa), respectively (Table 2). Liver MRI was performed on 43 available patients. The mean proton density fat fraction and magnetic resonance elastography values were 12.4% ± 7.8% (median: 11%, range: 1.5%-29.0%) and 2.6 ± 0.7 kPa (median: 2.4 kPa, range: 1.4-4.7 kPa), respectively (Table 2). Non-alcoholic steatohepatitis was diagnosed in 30 patients (48.4%). Patients with NASH had significantly higher HOMA index (*P = .*018), serum ALT (*P = .*002) and AST levels(*P = .*021), hepatic steatosis (*P = .*023), and fibrosis score (*P = .*001) than NAFL patients (Table 2).

### Cardiovascular Disease Risk

The mean ASCVD score was 7.5% ± 6.9% (median: 5.3%, range: 0.6%-34.8%). Of the 61 patients, 47.4% (n = 27) had low CVD risk, 15.8% (n = 9) had borderline CVD risk, whereas 36.8% (n = 21) had medium and high CVD risk. Coronary CT was performed on 53 patients. Coronary artery calcium was not detected in 69.8% of the patients (n = 37), whereas it was found in 30.2% of the patients (n = 16). Interestingly, 56.3% (9/16) of them had significant and extended atherosclerotic plaques (CAC > 100) (Table 3). Among the patients with medium-to-high ASCVD scores, 63.2% (12/19) of the patients had significant atherosclerotic plaque, and 21.1% (4/19) had extensive plaque burden (CAC > 400). The presence of NASH did not significantly affect CVR. No significant differences were found between NAFL and NASH patients in terms of the medium and high CVD risk (50% vs. 55.6%, *P = .*675) and the presence of CAC (40.7% vs.19.2%, *P = .*09).

### Cardiac Functions

Ventricular chamber and volume quantifications were within the normal limits in 52 NAFLD patients who were available for the measurements. The mean left atrial (LA) diameter was 38.6 ± 4.6 mm. The mean LV end-systolic and end-diastolic diameters were 29.3 ± 4.1 mm and 48.6 ± 3.8 mm, respectively. Left ventricular systolic function reflected by LV ejection fraction was 56.8% ± 5.8%. The presence of NASH did not significantly affect the LV systolic functions (56.8% ±7.0% vs. 57.2% ± 4.1%, *P = .*986). Cardiac output was 3.9 ± 0.9 L/min. Mean GLS, GCS, and GRS strains were (−14.4%) ± 3.5%, (−14.0%) ± 3.0%, and (38.8%) ± 9.3%, respectively. Mean area was (−24.5%) ± 5.1% (Table 4).

Non-alcoholic steatohepatitis was deleterious on LV diastolic functions. Mean mitral early and late velocities and E/A ratio were 71.4 ± 16.9 cm/s, 79.8 ± 17.2 cm/s and 0.93 ± 0.3. Mean A velocity in NASH patients was significantly increased compared to NAFL patients (87.0 ± 17.5 cm/s vs. 72.3 ± 13.6 cm/s, *P = .*002). The severity of fibrosis was significantly correlated with the A velocity (*P = .*019, *r* = 0.396). Mean E/e’ ratio was 8.1 ± 2.0. Of note, 44.2% (23/52) of the patients were undetermined (8-14). E/e’ ratio was slightly higher in patients with NASH compared to that of with NAFL patients (8.5 ± 2.5 vs. 7.6 ± 1.2, *P = .*125). Of note, 53% (n = 9) of the patients with medium-to-high risk as defined by their ASCVD scores presented diastolic dysfunction (E/e’ ratio > 8).

Cardiac MR was performed in available 43 NAFLD patients. Mean routine T1 and Siemens T1 values were 1016.8 ± 27.9 and 1000.9 ± 31.4, respectively. Submyocardial fibrosis was slightly more common in NASH patients than in NAFL patients (1003.8 ± 34.2 vs. 997.5 ± 28.4, *P = .*530).

## Discussion

The present study determined the association of NAFLD with subclinical coronary atherosclerosis and myocardial dysfunction in biopsy-diagnosed NAFLD patients. More than one-third of the asymptomatic NAFLD patients had a medium-to-high 10-year risk of CVD. Interestingly, 63% of them had significant atherosclerotic plaque. Also, 21% suffered from extensive plaque burden, which has been documented as an indication of CAD because of highly increased risk for a subsequent acute heart attack.^18^ The presence of CAC is a stronger predictor of ASCVD events than the CIMT.^26^ These findings confirm the results of a previous study that suggested that the presence of NAFLD was a CVD risk in asymptomatic NAFLD patients.^27^ The presence of NASH did not increase the CVD risk and the CAC scores in the present study. The findings indicate that NAFLD is associated with subclinical coronary atherosclerosis. This suggests that treatment decisions for CVD prevention should consider asymptomatic NAFLD patients with a medium-to-high 10-year risk of CVD with an elevated CAC score.

Several mechanisms could explain how NALFD increases the risk of developing CVD. Non-alcoholic fatty liver disease causes hepatic IR, altered lipid metabolism, hyperhomocysteinemia, increased oxidative stress, platelet activation, and endothelial dysfunction.^3,6-8^ Chronic inflammation increases endothelial dysfunction, alters the vascular tone, enhances the vascular plaque formation, and induces alterations to the cardiac structure and function.^3,6-8^ Non-alcoholic fatty liver disease is related to myocardial insulin resistance and impaired myocardial energy metabolism, resulting in the transformation of myocardial structures and functions.^28-32^ Cardiac structural changes occur at the early stages of NAFLD and can affect LV remodeling, increased cardiac mass, and diastolic dysfunction.^11-13^ Non-alcoholic fatty liver disease has been associated with several abnormal echocardiographic findings such as atrial and ventricular dilation or hypertrophy, LV systolic and diastolic dysfunction, subclinical myocardial dysfunction, and valvular calcifications.^11,12,33^ Canada et al^34^ found that NASH did not affect the resting diastolic parameters. However, hepatic fibrosis was significantly correlated with post-exercise E/e’ and, in turn, was significantly correlated with the fibrosis stage in stress echocardiography. At the early stages of diastolic dysfunction, resting LV pressures are normal. However, due to impaired relaxation, these patients can only increase stroke volume during exercise with an increase in filling pressure.^34^ Diastolic stress echocardiography could be more valuable in these patients to unmask any subclinical diastolic dysfunction.

Diastolic heart dysfunction is considered a clinically important predictor of mortality in individuals with preserved systolic function.^35^ Lee et al^11^ demonstrated that NAFLD patients had increased LV mass and mass index, LV end-systolic and end-diastolic diameters, and LA index compared to patients without NAFLD. The Coronary Artery Risk Developement in Young Adults (CARDIA) study demonstrated that NAFLD is longitudinally associated with subclinical LV remodeling, abnormal LV geometry, impaired LV function, and early diastolic dysfunction.^36^ The investigators concluded that obesity is the cause of the association between NAFLD and LV function.^36^ In contrast to previous studies, 2D and 3D speckle-tracking echocardiography was used in the present study. The ventricular chamber, volume quantifications, and LV EF were within the normal limits in patients with NAFLD. The presence of NASH did not significantly negatively affect LV systolic functions, while it was deleterious on LV diastolic functions. Among the NAFLD patients, 44% presented an undetermined E/e’ ratio referring to the increased LV filling pressure. E/e’ ratio was slightly higher in patients with NASH compared to that of NAFL patients (*P = .*125). Mean A velocity in patients with NASH was significantly increased compared to patients with NAFL (*P = .*002). The severity of fibrosis was significantly correlated with the A velocity (*r* = 0.396). Of note, more than half of the NAFLD patients (53%) with moderate-to-high 10-year CVD risk determined by their ASCVD scores presented diastolic dysfunction.

Subclinical systolic and diastolic dysfunction in NAFLD patients can be attributed to myocardial steatosis and probably, fibrosis.^11^ Previous limited studies with small samples demonstrated a significant relationship between the severity of NAFLD and LV systolic function in children and adults with NAFLD.^37,38^ In the CARDIA study, NAFLD participants presented deteriorating GLS over time, which was mostly attributed to obesity.^36^ In a recent study, patients with metabolic syndrome had a 2-fold increase in subclinical LV dysfunction risk if they had severe steatosis and a 1.7-fold increase if they had severe fibrosis.^37^ In the present study, the severity of fibrosis did not significantly correlate with diastolic dysfunction. However, NASH patients had numerically slightly lower 2D and 3D GLS levels than NAFL patients, which might suggest that subclinical LV dysfunctions begin at early stages, before liver fibrosis.

Although late-gadolinium-enhanced images are widely used to detect ischemia-induced cardiac fibrosis, a contrast difference between fibrotic and normal tissue is required to detect the abnormality.^15^ Therefore, it has serious limitations in detecting diffuse myocardial fibrosis.^15^ T1 mapping has been developed to overcome this problem.^15^ T1 values are used to obtain information about the international structure of the myocardium. It has been reported that in patients with aortic stenosis, native T1 values and the histologically determined collagen volume fraction are correlated.^39,40^ However, similar to our study, although native T1 values were higher in the NAFLD patient, no significant difference was observed among the NAFL and NASH patients, in a study conducted in patients with diabetic cardiomyopathy.^41^

In conclusion, based on the results of the present study, NAFLD seems to be associated with an increased risk of subclinical CVD and myocardial dysfunction in asymptomatic patients with cardiac disease. We suggest that the presence of NASH did not significantly increase the risk of CVD and subclinical myocardial dysfunction in such patients.

## Figures and Tables

**Table 1.** Baseline Characteristics of NAFLD Patients who Are Included in the Study

	All Patients (n = 61)	NAFL (n = 31)	NASH (n = 30)	*P*
Follow-up time, month				
Mean ± SD	21.3 ± 5.4	21.4 ± 5.5	21.1 ± 5.4	.919
Median	22	22	22.5	
Min-max	8-32	8-32	10-27	
Age				
Mean ± SD	53.1 ± 8.1	54.4 ± 7.8	51.7 ± 8.4	.205
Median	54	54	53.5	
Min-max	33-67	37-67	33-64	
Gender				
Female, n (%)	40 (65.6)	19 (47.5)	21 (52.5)	.474
Male, n (%)	21 (34.4)	12 (57.1)	9 (42.9)	
Comorbidities				
Diabetes, n (%)	26 (42.6)	10 (32.3)	16 (53.3)	.096
Hypertension, n (%)	22 (36.1)	12 (38.7)	10 (33.3)	.662
Hyperlipidemia, n (%)	13 (21.3)	8 (25.8)	5 (16.7)	.384
Smoking history, n (%)	9 (14.8)	3 (9.7)	6 (20)	.301
Insulin use, n (%)	6 (9.8)	2 (6.5)	4 (13.3)	.425
Metformin use, n (%)	22 (36.1)	8 (25.8)	14 (46.7)	.090
Statin use, n (%)	6 (9.8)	3 (9.7)	3 (10)	1.000
Systolic blood pressure, mmHg				
Mean ± SD	134.4 ± 16.5	132.6 ± 18	136.3 ± 14.7	.384
Median	131	128	133.5	
Min-max	104-178	104-178	110-166	
Diastolic blood pressure, mmHg				
Mean ± SD	79.9 ± 8.1	78.9 ± 9	80.9 ± 7	.351
Median	80	79	80	
Min-max	63-104	63-104	68-92	
Waist circumference, cm				
Mean ± SD	103.9 ± 11.0	103.6 ± 12.3	104 ± 9.6	.901
Median	103.0	103.0	102.5	
Min-max	82.0-129.0	82.0-129.0	86.0-118.0	
Hip circumference, cm				
Mean ± SD	111.5 ± 10.6	112.4 ± 12.1	110.4 ± 8.8	.447
Median	112.0	113.0	110.0	
Min-max	91.0-144.0	93.0-144.0	91.0-128.0	
Body mass index, kg/m^2^				
Mean ± SD	30.7 ± 4.4	31 ± 4.8	30.4 ± 4.0	.608
Median	30.4	30.8	30.0	
Min-max	19.6-43.1	19.6-43.1	23.9-39.4	
Body fat ratio (%)				
Mean ± SD	33.5 ± 8.1	33.3 ± 8.4	33.6 ± 7.8	.889
Median	34.9	35.3	34.85	
Min-max	15.3-49.6	15.3-49.6	16.6-45.1	
ALT, IU/L				
Mean ± SD	50.6 ± 40.6	36.1 ± 21.4	65.5 ± 50.0	.002
Median	41.0	28.0	53.5	
Min-max	6.0-215.0	6.0-89.0	12.0-215.0	
AST, IU/L				
Mean ± SD	38.7 ± 24.9	31.4 ± 17.1	46.1 ± 29.3	.021
Median	31.0	26.0	35.5	
Min-max	11.0-132.0	11.0-88.0	13.0-132.0	
GGT, IU/L				
Mean ± SD	57.3 ± 38.6	48.3 ± 30.5	66.5 ± 44	.089
Median	47.0	40.0	53.0	
Min-max	12.0-204.0	12.0-151.0	18.0-204.0	
Total bilirubin, mg/dL				
Mean ± SD	1.0 ± 1.4	1.0 ± 1.8	0.84 ± 0.5	.801
Median	0.7	0.7	0.7	
Min-max	0.3-2.7	0.4-2.7	0.3 -2.7	
Albumin, g/dL				
Mean ± SD	4.4 ± 0.5	4.3 ± 0.6	4.5 ± 0.3	.013
Median	4.4	4.3	4.5	
Min-max	1.7-5.2	1.7-4.8	3.8-5.2	
INR				
Mean ± SD	1.0 ± 0.1	0.95 ± 0.08	0.95 ± 0.07	.969
Median	0.95	0.95	0.95	
Min-max	0.8-1.2	0.77-1.18	0.82-1.12	
Creatinine, mg/dL				
Mean ± SD	0.7 ± 0.2	0.77 ± 0.16	0.67 ± 0.14	.012
Median	0.70	0.77	0.64	
Min-max	0.4-1.1	0.5-1.11	0.43-0.97	
Fasting blood glucose, mg/dL				
Mean ± SD	106.8 ± 24.9	112 ± 29.4	101.7 ± 18.7	.104
Median	98.0	101.5	95.0	
Min-max	77.0-196.0	78.0-196.0	77.0-147.0	
HbA1c, %				
Mean ± SD	6.5 ± 1.4	6.4 ± 1.4	6.5 ± 1.4	.863
Median	6.2	6.2	6.2	
Min-max	4.8-13.4	5.2-13.4	54.8-11.4	
Total cholesterol, mg/dL				
Mean ± SD	204.0 ± 39.5	206.4 ± 39.5	201.3 ± 39.8	.617
Median	197.0	207.0	195.0	
Min-max	130.0-315.0	130.0-315.0	130.0-278.0	
LDL, mg/dL				
Mean ± SD	127.3 ± 34.8	133.6 ± 37.6	120.7 ± 30.7	.150
Median	125.0	133.0	121.5	
Min-max	53.0-241.0	53.0-241.0	64.0-178.0	
TG, mg/dL				
Mean ± SD	167.7 ± 92.1	148.1 ± 46.4	187.9 ± 120.0	.609
Median	149.0	141.0	153.0	
Min-max	50-567	59-250	50-567	
HDL, mg/dL				
Mean ± SD	45.4 ± 9.3	44.9 ± 10.3	45.7 ± 8.1	.734
Median	46.0	45.0	46.5	
Min-max	27.0-66.0	27.0-66.0	32.0-64.0	
Uric acid, mg/dL				
Mean ± SD	5.5 ± 1.3	5.2 ± 1.2	5.7 ± 1.3	.140
Median	5.4	5.1	5.6	
Min-max	2.6-8.6	2.6-7.3	3.5-8.6	
Ferritin, mg/L				
Mean ± SD	83.7 ± 97.8	90.1 ± 120	77.0 ± 169.1	.931
Median	57.7	51.3	59	
Min-max	5.9-588.0	7.4-588.0	5.9-260	
CRP, mg/L				
Mean ± SD	4.9 ± 4.5	5.3 ± 5.4	4.4 ± 3.2	.636
Median	3.7	3.7	3.5	
Min-max	0.5-25.7	0.9-25.7	0.5-11.2	
Homocysteine, µmol/L				
Mean ± SD	8.9 ± 3.8	9.3 ± 3.8	8.5 ± 3.7	.506
Median	7.9	8.2	7.6	
Min-max	3.4-16.8	3.9-16.5	3.4-16.8	
Hemoglobin, g/dL				
Mean ± SD	14.1 ± 1.3	14.2 ± 1.2	13.9 ± 1.2	.451
Median	14.1	14.1	14.3	
Min-max	10.9-16.6	11.7-16.6	10.9-16	
Platelets, ×10^9^/dL				
Mean ± SD	271.1 ± 82.4	277.6 ± 76.5	264 ± 89	.538
Median	260	256	264	
Min-max	111-522	111-418	133-522	

NAFL, non-alcoholic fatty liver disease; NASH, non-alcoholic steatohepatitis; ALT, alanine aminotransferase; AST, aspartate aminotransferase; GGT, gamma-glutamyl transpeptidase; INR, international normalized ratio; HbA1c, glycated hemoglobin; LDL, low-density lipoprotein; TG, triglycerides; HDL, high-density lipoprotein; CRP, C-reactive protein; SD, standard deviation.

**Table 2.** Evaluation of Hepatic Steatosis and Fibrosis

	All Patients (n = 61)	NAFL (n = 31)	NASH (n = 30)	*P*
Fibrosis in biopsy, n (%)				.001
F0	23 (41.8)	16 (61.5)	7 (24.1)	
F1	18 (32.7)	7 (26.9)	11 (37.9)	
F2	7 (12.7)	3 (11.5)	4 (13.8)	
F3	7 (12.7)	0 (0)	7 (24.1)	
F4	0 (0)	0 (0)	0 (0)	
Fibroscan stiffness, kPa				
Mean ± SD	7.6 ± 5.3	6.7 ± 4.6	8.5 ± 6.0	.024
Median	6.0	5.3	7.6	
Min-max	3.5-34.7	3.5-26.3	4-34.7	
MR Elastography				
PDFF (n = 43)				
Mean ± SD	12.4 ± 7.8	9.9 ± 7.9	14.8 ± 7.0	.036
Median	11.0	6.8	12.0	
Min-max	1.5-29.0	1.5-29	5.6-28	
Stiffness, kPa (n = 41)				
Mean ± SD	2.6 ± 0.7	2.5 ± 0.5	2.7 ± 0.8	.318
Median	2.40	2.37	2.55	
Min-max	1.4-4.7	1.7-3.6	1.4-4.7	

NAFL, non-alcoholic fatty liver disease; NASH, non-alcoholic steatohepatitis; SD, standard deviation; MR, magnetic resonance; PDFF, proton density fat fraction.

**Table 3.** Cardiovascular Risk Assessment of Patients

	All Patients (n = 61)	NAFL (n = 31)	NASH (n = 30)
ASCVD Score (n = 57), n(%)			
Low risk (<5% risk), n (%)	27 (47.4)	15 (50)	12 (44.4)
Borderline risk (5%-7.5% risk), n (%)	9 (15.8)	4 (13.3)	5 (18.5)
Medium risk (7.5%-20% risk), n (%)	18 (31.5)	8 (26.7)	10 (37)
High risk (>20%), n (%)	3 (5.3)	3 (10)	0 (0)
Agatston Score (n = 53), n(%)			
0 (no calcification)	37 (69.8)	16 (59.3)	21 (80.8)
1-10 (minimum calcification)	1 (1.9)	1 (3.7)	0 (0)
11-100 (mild calcification)	6 (11.3)	6 (22.2)	0 (0)
101-400 (moderate calcification)	5 (9.4)	1 (3.7)	4 (15.4)
>400 (severe calcification)	4 (7.5)	3 (11.1)	1 (3.8)

NAFL, non-alcoholic fatty liver disease; NASH, non-alcoholic steatohepatitis.

**Table 4.** Echocardiography and Cardiac MR Assessments of Patients

	All Patients (n = 52)	NAFL (n = 26)	NASH (n = 26)	*P*
Aortic diameter, mm				
Mean ± SD	31.5 ± 3.8	32.5 ± 3.5	31.1 ± 3.7	.363
Median	32.0	33.0	32.0	
Min-max	24.0-39.0	26.0-39.0	24.0-37.0	
LA diameter, mm				
Mean ± SD	38.6±4.6	39.2 ± 4.5	38.8 ± 4.4	.834
Median	38.5	40.0	39.0	
Min-max	31.0- 50.0	31.0-50.0	32.0-45.0	
LV end-diastolic diameter, mm				
Mean ± SD	48.6±3.8	49.5 ± 2.9	48±4.1	.496
Median	48.5	49.0	48.0	
Min-max	41.0-57.0	45.0-56.0	41.0-57.0	
LV end-systolic diameter, mm				
Mean ± SD	29.3±4.1	29.6 ± 3.8	28.9±4.1	.687
Median	29.0	31.0	28.0	
Min-max	22.0-39.0	22.0-35.0	22.0-39.0	
IVS thickness, mm				
Mean ± SD	1.0±0.1	9.6 ± 1.1	10.2 ± 1.1	.05
Median	1.0	1.0	1.0	
Min-max	0.8–1.2	0.8-1.2	0.8-1.2	
Posterior wall thickness, mm				
Mean ±SD	1.0±0.1	0.9 ± 0.1	1 ± 0.1	.367
Median	1.0	0.9	1.0	
Min-max	0.8-1.2	0.8-1.1	0.8-1.2	
TAPSE, mm				
Mean ± SD	24.8±4.0	24.8 ± 4.5	24.6 ± 3.4	.814
Median	24	25	24	
Min-max	16-34	16-34	20-33	
Mitral PWE, cm/s				
Mean ± SD	71.4 ± 16.9	69.0 ± 13.7	73.6 ± 19.5	.337
Median	69	65	71	
Min-max	42-133	44–102	42–133	
Mitral PWA, cm/s				
Mean ± SD	79.8 ± 17.2	72.3 ± 13.6	87.0 ± 17.5	**.002**
Median	79	74	89	
Min-max	40-113	40-95	40-113	
PW Mitral E/A ratio				
Mean ± SD	0.92 ± 0.3	0.98 ± 0.29	0.86 ± 0.22	.141
Median	0.83	0.83	0.83	
Min-max	0.55-1.68	0.63-1.68	0.55-1.38	
PW Mitral DES, ms				
Mean ± SD	228.0 ± 34.7	226.0 ± 33.6	230.0 ± 36.2	.697
Median	222.5	216.5	236.5	
Min-max	156.0-300.0	176.0-287.0	156.0-300.0	
PW RV V, cm/s				
Mean ± SD	13.2 ± 4.0	13.04 ± 4	13.3 ± 3.4	.759
Median	13.0	13.0	13.0	
Min-max	6.0-24.0	6.0-20.0	8.0-18.0	
PW RV S, cm/s				
Mean ± SD	13.3 ± 2.7	13.0 ± 3	13.7 ± 2.5	.395
Median	13.0	13.0	13.0	
Min-max	6.0-19.0	6.0-17.0	10.0-19.0	
PW RV é, cm/s				
Mean ± SD	11.3 ± 3.0	11.21 ± 3.2	11.4 ± 3.0	.964
Median	11.0	11.0	11.0	
Min-max	5.0-18.0	5.0-17.0	7.0-18.0	
PW RV a’, cm/s				
Mean ± SD	15.8 ± 4.4	16.2 ± 3.9	15.2 ± 4.7	.345
Median	16.0	16.0	16.0	
Min-max	6.0-27.0	6.0-27.0	7.0-24.0	
PW RV MPI				
Mean ± SD	0.5 ± 0.2	0.46 ± 0.17	0.42 ± 0.14	.342
Median	0.43	0.47	0.41	
Min-max	0.1-0.9	0.14-0.85	0.16-0.70	
PW IVS S, cm/s				
Mean ± SD	7.4 ± 1.5	7.3 ± 1.7	7.6 ± 1.2	.570
Median	8.0	7.0	8.0	
Min-max	5.0-13.0	5.0-13.0	5.0-9.0	
PW IVS é, cm/s				
Mean ± SD	8.2 ± 2.2	8.4 ± 2.2	8.0 ± 2.1	.276
Median	8.0	8.0	8.0	
Min-max	5.0-13.0	5.0-13.0	5.0-13.0	
PW IVS a’, cm/s				
Mean ± SD	11.2 ± 4.5	11.8 ± 6.3	10.9 ± 2.3	.565
Median	10.5	10.0	11.0	
Min-max	7.0-40.0	8.0-40.0	7.0-17.0	
PW Lateral S, cm/s				
Mean ± SD	8.3 ± 2.6	8.4 ± 2.3	8.3 ± 2.4	.836
Median	8.0	9.0	8.0	
Min-max	5.0-14.0	6.0-15.0	5.0-14.0	
PW Lateral e’, cm/s				
Mean ± SD	10.0 ± 2.8	9.8 ± 2.3	10.1 ± 2.8	.883
Median	9.5	9.0	10.0	
Min-max	5.0-17.0	6.0-15.0	5.0-15.0	
PW Lateral a’, cm/s				
Mean ± SD	10.7 ± 3.3	10.7 ± 3.2	10.7 ± 3.5	.709
Median	10.5	10.0	11.0	
Min-max	5.0-22.0	6.0-20.0	5.0-22.0	
PW Lateral MPI				
Mean ± SD	0.5 ± 0.2	0.47 ± 0.15	0.47 ± 0.15	.879
Median	0.47	0.48	0.47	
Min-max	0.2-0.8	0.17-0.74	0.20-0.79	
AFI (2D), %				
Mean ± SD	(−16.8) ± 3.0	(−17.3) ± 2.8	(−16.2) ± 3	.190
Median	(−17)	(−18)	(−16.2)	
Min-max	(−11.9)-(−24.2)	(−12.3)-(−22.1)	(−11.9)-(−24.2)	
E/e’				
Mean ± SD	8.1±2.0	7.6 ± 1.2	8.5 ± 2.5	.125
Median	7.8	7.6	8.1	
Min-max	3.7-13.3	4.8-10.1	3.7 -13.3	
End-diastolic volume, mL				
Mean ± SD	95.7±20.0	95.1 ± 21.4	96.4 ± 19.6	.667
Median	91.0	93.0	91.0	
Min-max	58.0-147.0	58.0-147.0	70.0-145.0	
End-systolic volume, mL				
Mean ± SD	41.8±11.6	42.1 ± 13.4	41.4 ± 10.5	.903
Median	40.0	41.0	37.0	
Min-max	23.0-72.0	23.0-72.0	51.0-64.0	
Ejection fraction, %				
Mean ± SD	56.8±5.8	56.8 ± 7	57.2 ± 4.1	.986
Median	56.0	55.0	56.8	
Min-max	47.0-74.0	47.0-74.0	48.0-64.0	
Stroke volume, Ml				
Mean ± SD	54.1±10.6	53.1 ± 10.6	55.2 ± 10.7	.490
Median	52.5	52.0	54.0	
Min-max	35.0-81.0	35.0-76.0	38.0-81.0	
Cardiac output, L/min				
Mean ± SD	3.9±0.9	3.6±0.8	4.1±0.9	.058
Median	3.8	3.6	4.3	
Min-max	2.3-5.8	2.3-4.8	2.6-5.6	
End-diastolic mass, g				
Mean ± SD	111.4±10.1	109.17 ± 10.3	113.4 ± 9.9	.239
Median	112.0	109.0	114.0	
Min-max	91.0-130.0	91.0-126.0	91.0-130.0	
End-systolic mass, g				
Mean ± SD	111.4±9.9	109.8 ± 10.7	112.8 ± 9.4	.433
Median	111.5	109.0	114.0	
Min-max	92.0-130.0	92.0-130.0	93.0-128.0	
GLS, %				
Mean ± SD	(−14.4)±3.5	(−13.5) ± 4.1	(−15.1) ± 2.7	.189
Median	(−14.3)	(−12.5)	(−16.0)	
Min-max	(-8.0)-(−21.0)	(−8.0)-(−12.0)	(−11.0)-(−19.0)	
GCS, %				
Mean ± SD	(−14.0)±3.0	(−13.5) ± 3.0	(−14.4) ± 2.9	.402
Median	(−14.0)	(−13.0)	(−14.0)	
Min-max	(−8.0)-(−20.0)	(−8.0)-(−19.0)	(−10.0)-(−20.0)	
GRS, %				
Mean ± SD	38.8 ± 9.3	36.9 ± 10.1	40.2 ± 8.5	.301
Median	40.5	39.5	41.0	
Min-max	16.0-56.0	16.0-54.0	24.0-56.0	
Area strain, %				
Mean ± SD	(−24.5) ± 5.1	(−23.6) ± 5.7	(−25.2) ± 4.5	.351
Median	(−26.0)	(−25.5)	(−26)	
Min−max	(−33.0)-(−10)	(−10.0)-(−33.0)	(−16.0)-(−33.0)	
Rotation, %				
Mean ± SD	3.3 ± 4.2	3.3 ± 4.6	3.3 ± 3.9	.956
Median	2.75	2.7	3.9	
Min-max	(−3.0)-15.7	(−3.0)-15.7	(−2.0) -12.3	
Torsion, %				
Mean ± SD	1.0 ± 1.0	1.3 ± 0.75	0.81 ± 0.66	.401
Median	0.7	1.3	0.65	
Min-max	(−0.5)-5.0	(−0.5)-5.0	(−0.2)-2.1	
**Cardiac MR (n = 43)**				
Routine T1, ms				
Mean ± SD	1016.8±27.9	1013.3 ± 20.6	1019 ± 32.9	.455
Median	1009.0	1012.0	1006.2	
Min-max	976.3-1084.0	981.0-1050.0	976.0-1084.0	
Siemens T1, ms				
Mean ± SD	1000.9 ± 31.4	997.5 ± 28.4	1003.8 ± 34.2	.530
Median	1003.0	1001.7	1004.0	
Min-max	928.6-1077.0	928.0-1042.0	936.8-1077.0	

NAFL, non-alcoholic fatty liver disease; NASH, non-alcoholic steatohepatitis; SD, standard deviation; MPI, Myocardial performance index; LV, left ventricle; LA, left atrial; GLS, global longitudinal strain; GCS, global circumferential strain; GRS, global radial strain; MR, magnetic resonance; IVS, Interventricular septum; TAPSE, Tricuspid annular plane systolic excursion; PWE, Pulsed Wave E; PWA, Pulsed Wave A; DES, deceleration time.
